# The altruistic elderly, a valuable but unrecognised kidney donor group. A case report of an 85-year-old unspecified kidney donor

**DOI:** 10.1186/s12877-022-03511-8

**Published:** 2022-10-27

**Authors:** Okechukwu Okidi, Videha Sharma, Oana Piscoran, Fiona Biggins, Rajinder Singh, Titus Augustine

**Affiliations:** 1 The Manchester Centre for Transplantation, Manchester Royal Infirmary, Oxford Road, Manchester, M13 9WL UK; 2 Abdominal Organ Transplantation, Houston Methodist Hospital, Houston, TX 77030 USA; 3 Department of Renal Medicine, Lancashire Teaching Hospitals NHS Foundation Trust, Preston, UK; 4 Division of Diabetes, Endocrinology and Gastroenterology, Faculty of Medicine, Biology and Health, University of Manchester, Manchester Academic Health Science Centre, Manchester, UK

**Keywords:** Case report, Kidney transplant, Unspecified elderly kidney donor, Laparoscopic donor nephrectomy, Donor pool

## Abstract

**Background:**

Kidney transplantation is the definitive treatment for end stage renal disease (ESRD), offering improved quality of life and survival benefit over remaining on dialysis. There is, however, a prevailing significant mismatch between patients awaiting transplantation and available donor kidneys. Over time, initial stringent donor criteria have broadened and organs from extended criteria donors (ECDs) and older donors are now being accepted for transplantation. The spectrum of living donors has also undergone a change from close family members to an increasingly non-related, non-directed altruistic donors, newly classified as ‘unspecified’ donors. Unspecified elderly donors could be a potential untapped resource to expanding the kidney donor pool globally.

**Case Presentation:**

We present a case of an 85 year and 8 months old individual, who donated to an unrelated non-directed matched recipient in the national deceased donor transplant waiting list with excellent donor and recipient outcomes at 7 years.

**Conclusion:**

To our knowledge she is one of the oldest reported unspecified living kidney donors in the world to date. This case illustrates that elderly donors in good health can come forward to donate, knowing that it is safe and valuable. Once the immediate perioperative challenges after kidney donation are managed, elderly donors rarely encounter long term sequelae. We therefore report this case to increase awareness and refocus attention of transplant teams on elderly donors as a potential untapped group to help address the organ shortage problem in renal transplantation.

## Background

Kidney transplantation remains the preferred treatment for ESRD [1]. However, due to significant discrepancy between donor organ availability and recipient numbers, patients often remain on dialysis, on waiting lists or die before a suitable organ is identified. Globally organ shortage is being addressed by different strategies. With increasing experience and good donor outcomes, stringent criteria for donors have been relaxed and elderly donors who hitherto were thought unsuitable for donation are now being considered and accepted [1].

Elderly donors are generally classified as donors > 60 years and donation among carefully selected older individuals is associated with minimal perioperative risk and little or no added long-term renal dysfunction or ESRD [2]. Only few published reports exist of kidney donors above 80 years of age. The Guardian Newspaper in the United Kingdom (UK), on 17 May 2012, reported details of an 83 year old altruistic donor who at the time was the UK’s oldest living kidney donor [3]. Houston Methodist Hospital in Texas later reported the details of an 84-year-old man who wanted to help a neighbour with ESRD and, in the process, became the oldest living kidney donor in the United States (US) [4]. In July 2016 [5]. The Journal of Clinical Research reported an 87-year old grandfather who donated a kidney in a directed fashion to his grandson.

All these reported cases were uneventful with no perioperative complications, demonstrating feasibility, safety and good recipient outcomes with octogenarian donors. In this case report, we share our experience of an 85-year-old lady who donated her kidney altruistically as an unspecified donor to a recipient on the national kidney transplant waiting list with excellent outcome in both donor and recipient. To the best of our knowledge she is one of the oldest unspecified living kidney donors in the world to date.

## Case presentation

In 2013, a woman born in 1927 came forward to Lancashire Teaching Hospitals, one of our satellite referring centres expressing a desire to be an unspecified kidney donor. She was a retired teacher who in her own words, was healthy, had led a satisfactory life and was keen to contribute to society by this donation which would benefit someone on the national waiting list. The only significant past medical history was open fixation of right ankle fracture in 1990 and carpal tunnel decompression in 2009. Over several interviews with detailed discussions of risks of donor nephrectomy and long-term outcomes, she reiterated her full understanding of the risks and benefits and strong conviction to proceed. An independent psychological assessment confirmed full mental capacity and altruistic motive. The physical examination was normal. Her body mass index (BMI) was 31.2. An exhaustive donor work-up with additional cardiac tests in view of her age was completely satisfactory. An echocardiogram showed an ejection fraction of 74% and a myocardial perfusion scan was normal. A Tc99m-MAG3 isotopic scan confirmed excellent renal function with an isotopic GFR of 93 ml/min. The DMSA scan showed the left kidney contributing 46% and right 54% to overall renal function. The CT angiogram, for vascular anatomy demonstrated 2 renal arteries bilaterally with a normal urogram. Her case was then discussed by the multidisciplinary transplant team and a unanimous decision made not to disqualify her on basis of age as other parameters were entirely satisfactory. She was then registered on the United Kingdom Living Kidney Sharing Scheme (UKLKSS) as an unspecified donor and matched to a 53-year-old male recipient, with no potential living donors and listed on the deceased donor national waitlist, at a different centre. The UKLKSS is a highly successful scheme set up to benefit difficult to match patients on the deceased donor waiting list who don’t have living donors. Unspecified donors contributing to this scheme, provide opportunities for these recipients to have living donor (LD) transplants. The recipient had ESRD secondary to Polycystic Kidney Disease (PKD) and was 1–1-0 HLA mismatched with the donor. The donation operation was by a left-sided hand-assisted laparoscopic donor nephrectomy (LDN) lasting 1 h 58 min. There were no intraoperative problems. Post-operative care was with opioid based analgesia, early ambulation, incentive spirometry, laxatives and standard anti-thromboembolic prophylaxis with TED stockings and Dalteparin (5000iu) till discharge on day 4. The creatinine level at discharge was 93 μmol/L and eGFR 50 ml/min. Four weeks after discharge she had resumed driving and was back to near-normal routine and independence. On the most recent review her creatinine was documented to be 73umol/L with an eGFR > 60 ml/min. Currently, she is 94 years old and continues to lead an active, independent life and still continues to drive. She is aware of the successful recipient outcome anonymously, which has given her satisfaction with the donation and outcome and consents to sharing her experience in order to encourage other older donors to do same for the benefit to society.

**Fig. 1:**
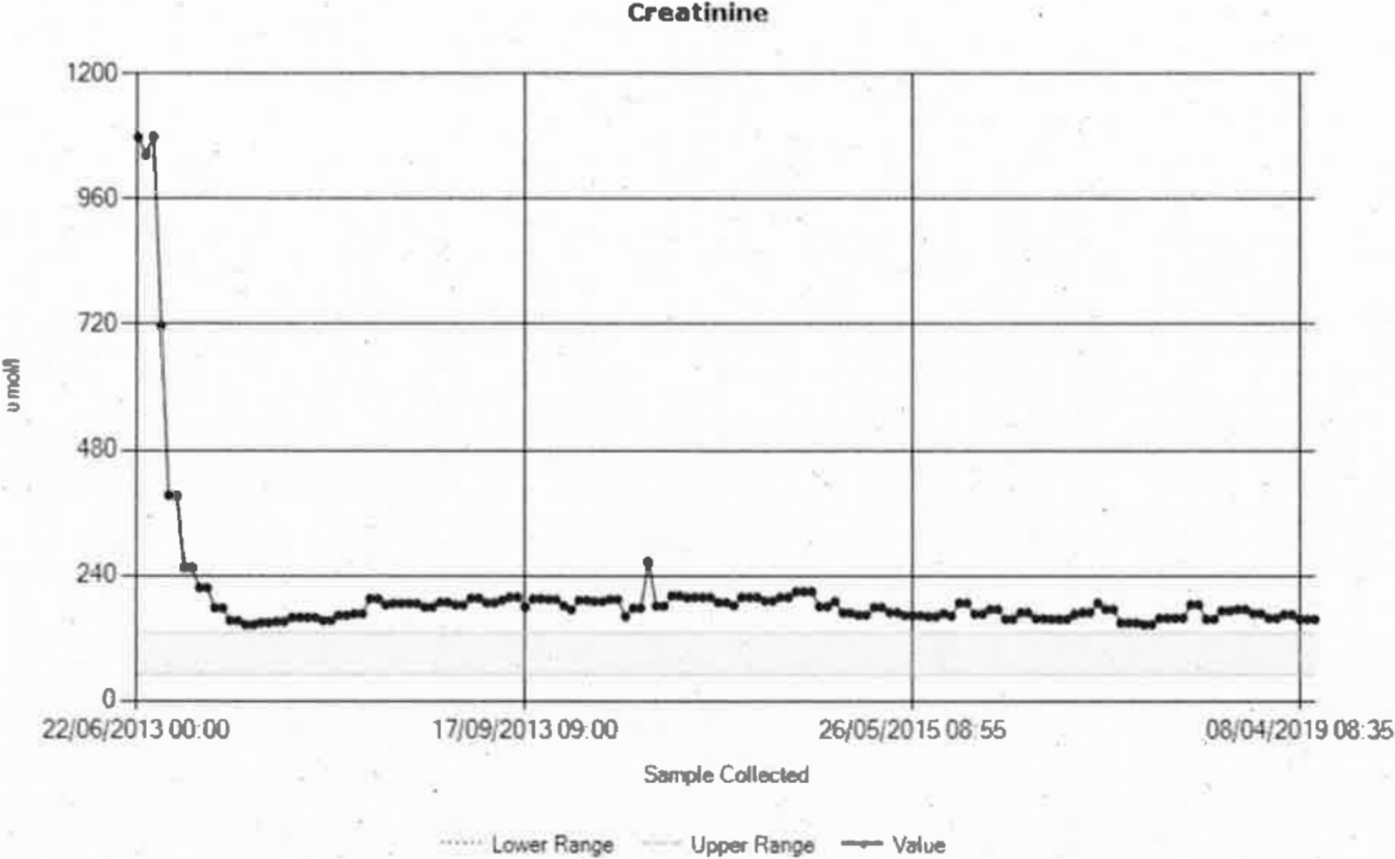
Creatinine trend in recipient post transplantation

**Fig. 2:**
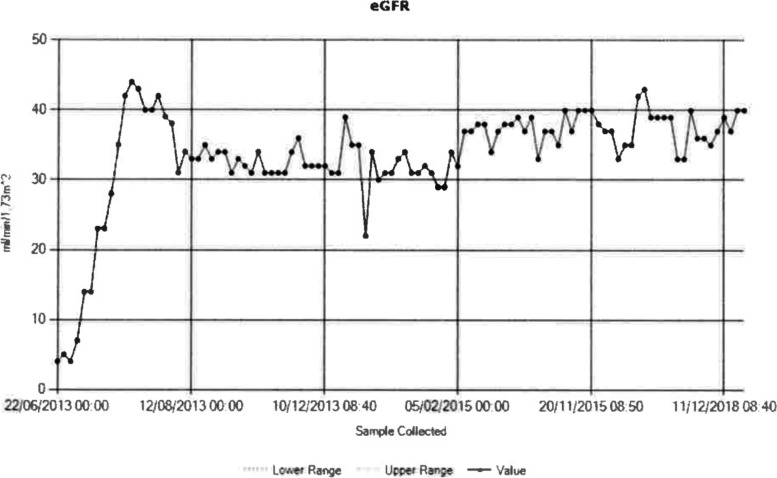
eGFR trend in recipient post transplantation

The kidney once retrieved was transported to the recipient centre and transplanted by 2 separate arterial anastomoses with a re-warm time of 30 min for the 1^st^ artery and 50 min for 2^nd^ artery; total ischemic time 5 h 26 min. The recipient achieved immediate graft function; and over last years has remained well with no rejection episodes, enjoying stable function, creatinine averaging 156 μmol/L and eGFR 40 ml/min (See Figs. 1 and 2).

## Discussion and conclusion

Kidney disease has a major effect on global health with 1.2 million people dying from Chronic Kidney disease (CKD) in 2017 [6]. Transplantation is considered the gold standard treatment for suitable individuals with advanced CKD. Kidney transplantation has come a long way since the first successful transplant between monozygotic twins carried out by Joseph Murray in 1954. That first transplant lasted eight years. Along with technical advances, there have been advances in immunosuppression and follow up of patients with vastly improved outcomes and graft lives. Whilst extremely successful, access to transplantation remains variable and insufficient to address global needs due mainly to shortage of suitable donor kidneys available to transplant [6]. Efforts to address the mismatch between donor kidney availability and demand in transplantation, has led to increasing utilization of marginal donors and ECDs [2]. In addition the ‘opt- out’ [7] policy in deceased organ donation is gaining popularity globally. The United Kingdom adopted donor ‘opt-out’ in 2019. While all these strategies have improved deceased donor organ numbers, living donor kidneys are considered the best quality organs, with the best recipient outcomes. They are carried out in an elective and planned setting with donor safety being the primary priority of all teams. Living donation has also transitioned from only close family members to unrelated and non-directed altruistic donors, newly classified as ‘unspecified’ donors [8]. With observed current trend of increasing ageing populations, an important potential donor group to consider in strategies to expand the donor pool is the unspecified elderly donor.

Older donors particularly, require thorough pre-operative evaluation and consideration of anaesthetic and surgical risks inherent with ageing. At the minimum, an echocardiogram, myocardial perfusion scan and pre-operative anaesthetic review should be done for all the donors. The use of the ESRD risk calculator tool [9] introduced in the US can support an empirical approach to donor selection, whereby multiple demographic and health characteristics are collated to estimate projected long-term risk of ESRD among living kidney-donor candidates and to inform acceptance criteria for donation. This calculator was not used in our donor but if applied will give her a score of 0.01% 15-year risk of ESRD.

Grams, et al.concluded that many older persons had low estimates of long-term ESRD risk even in the presence of health characteristics, such as low eGFR or mild hypertension, that are often considered contraindications to donation [9]. Data exist that indicate that a healthy > 60-year-old who successfully completes the rigorous donor medical work-up, has a comparable post donation long-term survival as would be expected of an advanced-age healthy individual who did not donate a kidney [2].

Many transplant centres use a ‘normal-for-now’ [10] standard for accepting young donors, in place of long-term risk estimates that must guide selection of all donors. An important factor to consider in these donors is life expectancy post donation. The younger the donor, the greater the remaining baseline life-time risk for ESRD, and the less ‘normal-for-now’ medical evaluation can foresee ESRD [10]. Long term risk of post-donation complications such as renal failure and hypertension is likely to be proportional to the remaining lifespan of the donor; it is therefore a strong argument for encouraging living donation from older donors.

The risk of hypertension post kidney donation remains controversial. Our patient had high systolic blood pressure of 150 – 160 mmHg by the 2^nd^ post-operative day but it settled with no anti-hypertensives. A meta-analysis by Boudville, et al.reported a 5-mm Hg increase in blood pressure within 5 to 10 years after donation over that anticipated with normal aging [11].

Another important parameter considered in donor selection is the BMI because higher BMIs are said to be more prone to complications [12, 13]. Our donor’s BMI was 31.2 but she had no perioperative complications. A systematic review and meta-analysis by L Franca [14] attested that LDN in obese donors with BMI > 30 is safe in terms of perioperative risk/ short term outcomes. The ultimate decision on BMI depends on the experience of donor teams and individual surgeon.

Hourmant et al. [15] reported post donation eGFR < 60 ml/min in 80% of older donors compared to 31% of younger donors. This was not reflected in our index patient whose current eGFR is > 60 ml/min. It can be argued that lower GFR post donation in elderly donors seems to be more of a phenomenon of aging rather than sequelae of donation. With normal aging, nephron loss occurs and is reflected in the age-related decrease in GFR [16]. However, 50% of older adults don’t have a significant decline in kidney function [7].

From the recipients’ perspective, living donation even from elderly donors provides shorter wait times, [17] less dialysis exposure and expands the overall pool of donor organs thereby benefitting all transplant candidates. Furthermore, older living donor allografts have survival rates similar to or better than any deceased donor allograft, even in the setting of poor HLA matching [17]. The recipient of our patient’s kidney has continued to maintain stable graft function 7 years after transplantation in spite of a 32-year age difference, clearly highlighting the specific benefit of a living donor kidney.

Our donor on a recent annual review continues to express satisfaction with her outcome, with good incisional scars and no herniae, confirming Klop et al.’s findings that elderly donors have better cosmetic outcome after LDN [18]. There were no psycho-social issues expressed at the review; she expressed no regrets with her decision to donate and actually consented to this case report to encourage other potentially elderly donors. Bhatta et al. have reported on altruistic attitudes among older adults [19] and Sparrow et al. have conducted a metaanalysis on aging and altruism [20]. Bhatta et al. state that these altruistic prosocial attitudes can be useful for planning programs and policies that utilise older adults’ potential for making contributions to society. Recognising older adults generosity of spirit and desire to help others can also counteract ageist attitudes. The example demonstrated by our donor encapsulates all aspects described in these two references and which can potentially be explored as a strategy to increase the organ donor pool.

For transplant programs these good outcomes should alleviate concerns surrounding safety of utilization of kidney from older donors both from the donor and recipient perspectives.

It is hence disappointing therefore that despite excellent outcome in recipients of kidneys from older donors, [11] utilization of older donors remains low currently. For instance, only 2.8% of living kidney donors in the US were 65 years of age or older in 2014 [21].

The reason for this primarily may be the lack of awareness that donation from the elderly is safe and valuable. Burnapp, et al. have recommended raising awareness and engaging select target audiences to encourage unspecified kidney donations in order to increase transplant opportunities for patients with ESRD [22]. Furthermore, a kidney donated by an unspecified donor can be a trigger for chain donations permitting multiple transplants. In the US, a kidney chain resulted in 30 transplants occurring from one single non-directed living kidney donor [23]. The UK Living Donor Strategy has a set goal for 75% of altruistic donations to be used for chains with three transplants in each chain from year 2020 (https://www.organdonation.nhs.uk).

In addition, an unspecified living donor can enable pre-emptive transplantation or provide elective nature for donor/ recipient surgeries.

This case report of an 85-year-old living unspecified kidney donor, demonstrates excellent outcomes in both donor and recipient. It adds to a limited number of similar reports and seeks to refocus attention to older donors as a potential untapped group for increasing donor availability to address the increasing numbers of ESRD patients on transplant waiting lists. It also reinforces the need for professionals to disseminate the fact that older individuals in relatively good health can donate in a non-directed fashion and may trigger a chain donation thereby benefiting multiple recipients, difficult – to—match patients on the deceased donor waitlist who do not have LDs and will get LD kidney transplants and those with incompatible living donor pairs. Though the average cold ischemia time (CIT) is more prolonged with the kidney exchange schemes where kidney travel rather than donors, overall graft function remains at par with non – kidney exchange transplantation [24].

Age alone taken as an individual factor is not a contraindication to kidney donation. While this is only a case report with its inherent limitations, it signposts transplant teams to a potential untapped source of healthy older individuals in society who may be prepared to donate in an altruistic non—directed fashion for the benefit of ever-increasing ESRD patients on transplant waiting lists the world over.

## Data Availability

The authors declare that data supporting the findings of this study are available within the article and its supplementary information files.

## Abbreviations

BMI: Body mass index
CIT: Cold Ischemia Time
CKD: Chronic kidney disease
CT: Computed tomography
DMSA: Di mercapto succinic acid
ECD: Extended criteria donors
ELPAT: Ethical, legal and psychosocial aspects of organ transplantation
ESRD: End stage renal disease
GFR: Glomerular filtration rate
HLA: Humal leukocyte antigen
MAG3: Mercapto acetyl triglycine
PKD: Polycystic kidney disease
Tc 99 m: Technetium 99 metaisomer
TED: Thrombo embolus deterrent
UK: United Kingdom
UKLKSS: United Kingdom living kidney sharing scheme
US: United States
